# A dynamic time‐to‐event model for prediction of acute graft‐versus‐host disease in patients after allogeneic hematopoietic stem cell transplantation

**DOI:** 10.1002/cam4.6833

**Published:** 2023-12-22

**Authors:** Katharina Och, Amin T. Turki, Katharina M. Götz, Dominik Selzer, Christian Brossette, Stefan Theobald, Yvonne Braun, Norbert Graf, Jochen Rauch, Kerstin Rohm, Gabriele Weiler, Stephan Kiefer, Ulf Schwarz, Lisa Eisenberg, Nico Pfeifer, Matthias Ihle, Andrea Grandjean, Sonja Fix, Claudia Riede, Jürgen Rissland, Sigrun Smola, Dietrich W. Beelen, Dominic Kaddu‐Mulindwa, Jörg Bittenbring, Thorsten Lehr

**Affiliations:** ^1^ Department of Clinical Pharmacy Saarland University Saarbrücken Germany; ^2^ Department of Hematology and Stem Cell Transplantation, West‐German Cancer Center University Hospital Essen Essen Germany; ^3^ Department of Pediatric Oncology and Hematology Saarland University Homburg Germany; ^4^ Department of Biomedical Data & Bioethics Fraunhofer Institute for Biomedical Engineering (IBMT) Sulzbach Germany; ^5^ Institute for Formal Ontology and Medical Information Science Saarland University Saarbrücken Germany; ^6^ Department of Computer Science University of Tübingen Tübingen Germany; ^7^ Averbis GmbH Freiburg Germany; ^8^ Institute of Virology Saarland University Medical Centre Homburg Germany; ^9^ Helmholtz Institute for Pharmaceutical Research Saarland (HIPS), Helmholtz Centre for Infection Research (HZI) Saarland University Campus Saarbrücken Germany; ^10^ Department of Internal Medicine 1 University Hospital of the Saarland Homburg Germany

**Keywords:** acute graft‐versus‐host disease, allogeneic hematopoietic stem cell transplantation, cyclosporine a, risk factors, time‐to‐event model

## Abstract

**Background:**

Acute graft‐versus‐host disease (aGvHD) is a major cause of death for patients following allogeneic hematopoietic stem cell transplantation (HSCT). Effective management of moderate to severe aGvHD remains challenging despite recent advances in HSCT, emphasizing the importance of prophylaxis and risk factor identification.

**Methods:**

In this study, we analyzed data from 1479 adults who underwent HSCT between 2005 and 2017 to investigate the effects of aGvHD prophylaxis and time‐dependent risk factors on the development of grades II–IV aGvHD within 100 days post‐HSCT.

**Results:**

Using a dynamic longitudinal time‐to‐event model, we observed a non‐monotonic baseline hazard overtime with a low hazard during the first few days and a maximum hazard at day 17, described by Bateman function with a mean transit time of approximately 11 days. Multivariable analysis revealed significant time‐dependent effects of white blood cell counts and cyclosporine A exposure as well as static effects of female donors for male recipients, patients with matched related donors, conditioning regimen consisting of fludarabine plus total body irradiation, and patient age in recipients of grafts from related donors on the risk to develop grades II–IV aGvHD. Additionally, we found that higher cumulative hazard on day 7 after allo‐HSCT are associated with an increased incidence of grades II–IV aGvHD within 100 days indicating that an individual assessment of the cumulative hazard on day 7 could potentially serve as valuable predictor for later grades II–IV aGvHD development. Using the final model, stochastic simulations were performed to explore covariate effects on the cumulative incidence over time and to estimate risk ratios.

**Conclusion:**

Overall, the presented model showed good descriptive and predictive performance and provides valuable insights into the interplay of multiple static and time‐dependent risk factors for the prediction of aGvHD.

## 1 INTRODUCTION

Acute graft‐versus‐host disease (aGvHD) is a major cause of death for patients after allogeneic hematopoietic stem cell transplantation (HSCT).^1^, ^2^ Despite therapeutic advancements in the field of HSCT over the last decades,^3^ treating moderate to severe aGvHD (grades II–IV) is especially challenging in steroid‐refractory disease. Hence, rational use of available prophylaxis and identification of risk factors for the development of aGvHD are crucial.

Previous studies on aGvHD risk factors consistently reported that human leukocyte antigen disparity,^4^, ^5^, ^6^ female donors for male recipients,^4^, ^6^, ^7^ patient age,^4^, ^5^, ^8^, ^9^, ^10^ prior alloimmunization of the donor,^4^, ^7^, ^10^ and insufficient GvHD prophylaxis^4^, ^5^ increases the risk for development of grades II–IV aGvHD. Other risk factors, such as donor age,^4^, ^11^ intensity of the conditioning regimen,^4^, ^12^ or stem cell source^4^, ^8^, ^12^ are discussed more controversially. Current standard aGvHD prophylaxis consists of a combination of methotrexate with a calcineurin inhibitor, cyclosporine A (CsA; predominantly used in Europe) or tacrolimus, or a combination of CsA with mycophenolate mofetil.^13^ Previous investigations revealed a significant relationship between low CsA blood levels and higher incidences of aGvHD after HSCT.^14^, ^15^, ^16^, ^17^, ^18^, ^19^, ^20^, ^21^ Alternative prophylaxis strategies aim to remove or modulate donor T‐cells ex‐vivo or in‐vivo by monoclonal or polyclonal antibodies,^4^, ^22^ such as anti‐thymocyte globulin (ATG). Especially for patients with matched unrelated donors, ATG complements standard GvHD prophylaxis to prevent aGvHD and chronic GvHD,^23^ although treatment success for prophylaxis of aGvHD varies.^22^ Despite prophylaxis and current knowledge of risk factors, approximately 40% of HSCT recipients develop moderate to severe aGvHD.^4^, ^14^, ^16^, ^17^ Most risk factor studies identified static variables, overlooking the highly dynamic interindividual variability of the post‐HSCT process.

Thus, static and baseline variables alone may be insufficient for individual risk assessment of aGvHD development and course. Dynamic models with longitudinal, time‐dependent data could improve risk prediction^24^, ^25^ and help monitor prophylaxis effects on grades II‐IV aGvHD. Hence, this study focused on the development and application of a dynamic longitudinal time‐to‐event (TTE) analysis for the time from HSCT until the initial diagnosis of aGvHD to investigate prophylaxis effects and time‐dependent risk factors for the development of harmful grades II–IV aGvHD in a large single‐center patient cohort.

## 2 METHODS

### 2.1 Clinical data

Data were derived from the XplOit study, where an ontology‐based IT platform was developed to harmonize and pseudonymize large quantities of heterogeneous data from hospital information systems for the development of predictive models in the field of stem cell transplantation.^26^ Clinical data were retrospectively collected from 1783 adult patients who had undergone allogeneic HSCT between January 2005 and August 2017 in the Department of Hematology and Stem Cell Transplantation of the West‐German Cancer Centre at University Hospital Essen. Ethics approvals were obtained from the institutional review board (IRB) of the medical association of the Saarland (Protocol N° 33/17) and the IRB of the University Duisburg‐Essen (Protocol N° 17‐7576‐BO). The requirement for written informed consent was waived due to the retrospective nature of the research and the use of anonymized data.

### 2.2 Dataset generation

The endpoint of this study was time to the first diagnosis of aGvHD in patients developing maximum grades II–IV aGvHD after HSCT. The dataset included the time of initial diagnosis of aGvHD and the maximum overall grades of severity within 100 days post‐HSCT. The overall grade of severity was calculated using the Consensus aGvHD Grading criteria^27^ based on the maximum recorded organ stages. The initial grade of severity was not recorded. Only patients without multiple HSCTs (without history of previous transplantation or subsequent transplantation until 2019) and with a plausible date of aGvHD diagnosis (recorded after HSCT and prior death), which received CsA as aGvHD prophylaxis and with available laboratory data, were included in this analysis. To estimate the effect of CsA, only patients with at least one CsA measurement per week on average and at least one measurement in the week after HSCT and the week before diagnosis or censoring were included (Figure S1). The dataset comprised 1479 eligible patients, randomly assigned to training and test datasets in a 3:1 ratio. Right censoring was performed for dropouts (Type I^28^) and at day 100 after HSCT.

### 2.3 Data analysis

**FIGURE 1:**
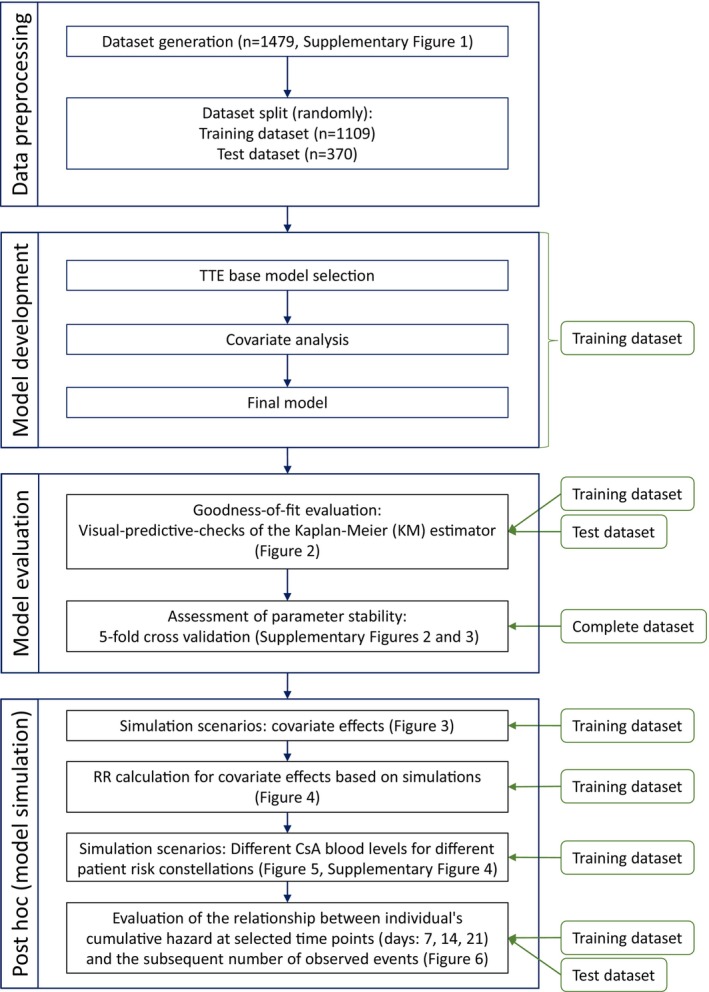
Analysis plan flow chart. The left side displays the primary and, in the center, blue boxes outline detailed steps. On the right, green rounded boxes indicate the datasets used for each corresponding analysis step. RR, risk ratio.

NONMEM® (version 7.4.3, ICON Development Solutions, Ellicott City, MD, USA)^29^ was used for TTE modeling, with the Laplacian method used for parameter estimation.^30^ Visual Predictive Checks (VPCs) were simulated via Pearl‐speaks‐NONMEM® (version 4.8.1).^31^ Model selection was based on significant changes in the NONMEM® objective function value (OFV; *p*‐value <0.05), precision of parameter estimation (relative standard error (RSE) < 50%), and VPC inspection. VPC simulations used 1000 dataset replicates to assess observed versus model‐predicted 95% confidence intervals (CI). R (version 3.6.3, The R Foundation for Statistical Computing, Vienna, Austria) was used for visualization and statistical evaluation. In addition to the evaluation of the final model performance on the retained test dataset, a five‐fold cross validation was performed to address bias, potential overfitting, and parameter stability (for detailed information see Supplementary Methods 1.2 Cross validation). The analysis plan, along with subsequent post hoc steps, is illustrated in Figure 1.

### 2.4 Model development

The parametric survival function, as shown in Equation 1, was used to analyze the time to the first diagnosis of grades II–IV aGvHD.

(1) $$S\left(t\right)=e^{-\int_{0}^{t}h\left(t\right)\mathrm{dt}}$$

*h(t)* = Hazard function for aGvHD onset, *S(t)* = Probability of not having a diagnosis of grades II–IV aGvHD within the time interval 0 (transplantation) to time *t*.

A base model was developed testing different functions for h(t) including proportional, Weibull and Gompertz approaches as well the Bateman function as non‐monotonic hazard descriptions^32^ with and without a time delay for hazard onset (Table 1).

**TABLE 1:** Tested hazard functions with resulting objective function values (OFVs).

Trend	Description	Equation (Hazard function)	OFV
Monotonic	Proportional	$h\left(t\right)=\lambda$	4814.305
	Weibull	$h\left(t\right)=\lambda *\alpha *{\left(\lambda *t\right)}^{\alpha -1}$	4780.307
	Gompertz	$h\left(t\right)=\lambda *e^{\alpha *t}$	4813.129
Non‐monotonic	Bateman	$h\left(t\right)=f*\frac{\mathrm{ka}}{\mathrm{ka}-\mathrm{ke}}\left(e^{-\mathrm{ke}*t}-e^{-\mathrm{ka}*t}\right)$	4618.129
	Bateman hazard + Time delay	As given in the NONMEM control file provided in supplementary	4533.434

Abbreviations: f, scale parameter; ka, shape parameter (absorption), ke, shape parameter (elimination); t, time, λ, scale parameter; α, shape parameter.

### 2.5 Covariate model

Relevant covariates were obtained from previous literature^4^, ^5^, ^6^, ^7^, ^9^, ^10^, ^11^, ^12^, ^33^ or identified by exploratory analysis of covariate‐stratified Kaplan–Meier curves using the existing dataset (Table S1). Multivariable analysis was performed on the base model (significance level of 5%) using longitudinal data of cell counts and CsA blood levels as well as static data of patient characteristics, donor characteristics, and HSCT procedure. Covariate effects were either implemented by directly modulating the base hazard or the hazard function parametrization. Continuous covariates were centered around the population median and tested via linear or power functions. Missing values were imputed as described in the supplement. CsA whole blood concentrations obtained during clinical routine were quantified via antibody‐conjugated magnetic immunoassay (ACMIA). To avoid confounding effects of other immunosuppressive drugs, patients that switched from CsA prophylaxis to another immunosuppressive drug were censored at the last time of CsA measurement.

### 2.6 Conditioning regimen, GvHD prophylaxis and HLA‐typing

Different conditioning regimens were employed, including various drug combinations (23% fludarabine, 22% fludarabine/treosulfan, 17% busulfan/fludarabine) with or without total body irradiation (TBI; Table S2). Fludarabine was always combined with TBI (2–12 Gy, FluTBI). The effects of drug combinations and the use of TBI were analyzed separately.

GvHD prophylaxis consisted predominantly of CsA plus methotrexate (82%, Table S3). In‐vivo T‐cell depletion by ATG was assigned additionally to approximately 55% patients based on clinical treatment protocols (Table S4–S6). Treatment of grade I aGvHD adhered to standard procedures without systemic treatment or intensification of CsA blood levels.

HLA testing was performed using high‐resolution typing. Donors with a 10/10 HLA matching at HLA‐A, ‐B, ‐C, ‐DRB1, ‐DQB1 were categorized as “matched donors”, whereas 9/10 and lesser matches were classified as “unmatched donors”. It should be noted that HLA‐DPB1 was not factored into the assessment of donor‐recipient compatibility.

### 2.7 Stochastic simulations

Upon completion of model development, stochastic simulations were performed with the final model. For this, the final model was used to simulate 1000 replicates of the training dataset to assess the effect of covariates on the cumulative incidence of grades II‐IV aGvHD over 100 days after HSCT. Each covariate effect was explored individually while keeping other covariates constant. Risk ratios (RRs) were calculated as described in the supplement to investigate the impact of covariates on the development of grades II–IV aGvHD within 100 days posttransplantation.

## 3 RESULTS

### 3.1 Patient characteristics

The final dataset included 1479 eligible study patients, who were randomly assigned to either the training dataset (*n* = 1109) or the test dataset (*n* = 370). Patient characteristics were comparable between both datasets (Table 2, *p* ≥ 0.05). Patients (median age 54 years, 57% male) were most often diagnosed with acute myeloid leukemia (46.5%). Donors (median age 37 years) were primarily matched unrelated donors (MUD; 53%). Within 100 days and 1 year after HSCT, 142 (9.6%) and 301 (20.3%) relapses as well as 156 (10.5%) and 526 (35.5%) deaths occurred, respectively. The cumulative incidence of grades II–IV aGvHD was 41% within 100 days after HSCT for all patients. Since patients that switched from CsA prophylaxis to another immunosuppressive drug were censored at the last time of CsA measurement, the analysis included 565 aGvHD patients (38%, grade II: *n* = 467, grade III: *n* = 89, grade IV: *n* = 9) with a median time to onset of 20 days. Median CsA blood concentration was 178 ng/mL (5th–95th percentile: 48.0–333.1 ng/mL) within 100 days after HSCT are were recorded on average (median) until day 91 (5th–95th percentile: 24–100 days). The complete dataset included 68,109 measurements of white blood cells (WBC), 41,469 measurements of lymphocytes, and 32,959 CsA blood concentrations within 100 days after HSCT. For parameter estimation, training dataset records were used up to an event or censoring, containing 33,285 WBC counts, 17,860 lymphocyte counts, and 17,237 CsA blood concentrations.

**TABLE 2:** Patient Characteristics.

	Training (*n* = 1109)		Test (*n* = 370)		*p* ^a^	Complete (*n* = 1479)	
	Median	Range	Median	Range		Median	Range
Patient age (years)	54	17–76	53.5	17–74	0.423	54	17–76
Donor age (years)	37	12–80	37	13–70	0.957	37	12–80
Months between diagnosis and HSCT	9	1–412	9	2–205	0.812	9	1–412
Death after HSCT (days)	265	1–4896	216	7–3867	0.152	249.5	1–4896
Relapse after HSCT (days)	147	11–4048	189	15–2539	0.206	156	11–4048

		Count	%	Count	%		Count	%
Recipient sex	Male	641	57.8	201	54.32	0.250	842	56.93
	Female	468	42.2	169	45.68		637	43.07
Donor sex	Male	713	64.29	247	66.76	0.414	960	64.91
	Female	396	35.71	123	33.24		519	35.09
Female‐to‐male transplantation		159	14.34	49	13.24	0.666	208	14.06
Donor type^b^	Matched unrelated	595	53.65	194	52.43	0.895	789	53.35
	Matched related	255	22.99	89	24.05		344	23.26
	Unmatched unrelated	247	22.27	83	22.43		330	22.31
	Unmatched related	12	1.08	4	1.08		16	1.08
Stem cell source	Bone marrow (BM)	87	7.84	27	7.3	0.323	114	7.71
	Peripheral blood stem cell (PBSC)	1014	91.43	343	92.7		1357	91.75
	BM and PBSC	8	0.72	0	0		8	0.54
Diagnosis	Acute myeloid leukemia	516	46.53	171	46.22	0.126	687	46.45
	Acute lymphoblastic leukemia	106	9.56	46	12.43		152	10.28
	Myelodysplastic syndromes	103	9.29	39	10.54		142	9.6
	Myeloproliferative Disorder	71	6.4	29	7.84		100	6.76
	Chronic myeloid leukemia	51	4.6	24	6.49		75	5.07
	Non‐Hodgkin's lymphoma	113	10.19	32	8.65		145	9.8
	Others^c^	149	13.43	29	7.83		178	4.06
Disease stage before HSCT^d^	Early	341	30.75	126	34.05	0.352	467	31.58
	Advanced	616	55.55	201	54.32		817	55.24
	Unknown	152	13.71	43	11.62		195	13.18
Conditioning regimen	Fludarabine + Treosulfan	231	20.83	91	24.59	0.244	322	21.77
	Busulfan + Fludarabine	194	17.49	61	16.49		255	17.24
	Fludarabine + TBI (8 Gy)	132	11.9	44	11.89		176	11.9
	Fludarabine + TBI (12 Gy)	60	5.41	21	5.68		81	5.48
	Fludarabine + TBI (10 Gy)	53	4.78	22	5.95		75	5.07
	Carmustine + Fludarabine + Melphalan	102	9.2	18	4.86		120	8.11
	Cyclophosphamide + TBI (12 Gy)	51	4.6	24	6.49		75	5.07
	Cyclophosphamide + TBI (10 Gy)	40	3.61	12	3.24		52	3.52
	Busulfan + Cyclophosphamide + Fludarabine	57	5.14	11	2.97		68	4.6
	Etoposide + TBI (12 Gy)	36	3.25	25	6.76		61	4.12
	Others^e^	153	13.8	41	11.08		194	13.12
ATG	Yes	612	55.18	200	54.05	0.718	812	54.9
Recipient CMV serostatus	Positive	640	57.71	210	56.76	0.709	850	57.47
	Negative	459	41.38	155	41.89		614	41.51
	Unknown	10	0.90	5	1.35		15	1.01
Relapse		311	28.04	103	27.84	1.00	414	27.99
Graft loss		9	0.81	2	0.54	0.741	11	0.74
Acute GvHD	Total	887	79.98	305	82.43	0.635	1192	80.59
	Grade 1	434	39.13	145	39.19		579	39.15
	Grade 2	370	33.36	128	34.59		498	33.67
	Grade 3	77	6.94	28	7.57		105	7.1
	Grade 4	6	0.54	4	1.08		10	0.68
Chronic GvHD	De‐novo	97	8.75	27	7.3	0.244	124	8.38
	Progressive	577	52.03	211	57.03		788	53.28
	No chronic GvHD	435	39.22	132	35.68	0.150	567	38.34
cGvHD Stadium	Limited	384	34.63	149	40.27		533	36.04
	Extensive	290	26.15	89	24.05		379	25.63

^a^ Unadjusted *p*‐values of Fisher's exact test (if feasible), χ^2^ test or two‐sample Wilcoxon test.

^b^ For donor‐recipient matching human leukocyte antigen (HLA)‐A, ‐B, ‐C, ‐DRB1, ‐DQB1 were considered.

^c^ Other diagnosis includes the following malignancies: Hemoglobinopathy, Congenital hematologic disorder, Other hematologic malignancies, Chronic myelomonocytic leukemia, Hodgkin's lymphoma, Non‐malignant hematological diseases, Chronic lymphocytic leukemia, Multiple Myeloma.

^d^ Early stages: De‐novo AML in 1st remission, ALL in 1st remission, MDS with single lineage dysplasia, and MDS with single lineage dysplasia and ring sideroblasts, CML in 1st chronic phase. Advanced disease stages: All other stages that did not correspond to early stages, such as AML in 2nd remission.

^e^ A detailed list of all conditioning regimens is given in Table S2.

### 3.2 aGvHD model

Among all tested hazard functions, the non‐monotonic Bateman function provided the best description of the time‐varying hazard of grades II–IV aGvHD. The analysis revealed a delayed onset of hazard with a mean transit time of 11.3 days (*p* < 0.001). The maximum hazard was observed after 17 days (population median) and it declined with a half‐life of 11.3 days. Multivariable covariate analysis revealed WBC count, female donors for male recipients, matched related donor (MRD), FluTBI conditioning regimen, CsA exposure, and patient age as significant covariates (*p* < 0.05).

WBC count was included as a continuous time‐dependent covariate. Here, a lower WBC count was associated with lower hazards, with an estimated exponent of 0.125 using an exponential model centred around the daily population median WBC count. In the study population, the WBC count followed a typical trajectory, with a decrease in the first few days after transplantation (minimum: 0.055 cells/nl on day 8), followed by an increase (maximum: 6.0 cells/nl on day 41) and subsequent stabilization close to WBC reference values (3.9 cells/nl (median) within day 50–100). As the daily population median WBC count varied over time, individual WBC counts affected hazards over time differently. For example, a decrease in WBC count by 2.0 cells/nl from the median WBC count of 2.3 cells/nl and 4.7 cells/nl on day 20 and day 30 reduced the hazard by 22.3% and 6.6%, respectively. Neither a significant effect of lymphocytes, nor granulocytes nor the ratio of lymphocytes over granulocytes could be detected on the hazard rate.

The hazards for male recipients with female donors were estimated to be 1.45 times higher than for other donor‐recipient gender combinations. Patients treated with FluTBI had a hazard reduction of 23.2% compared to patients treated with other drug combinations, and patients with MRD had a hazard reduction of 22.7% compared to patients with MUD or unmatched donors.

CsA exposure as a continuous time‐depending covariate significantly affected hazard elimination. A linear model centred around 250 ng/mL CsA and an estimated slope of 0.00309 described this relationship, indicating that patients with higher CsA levels had a more rapid decrease in hazard. For instance, assuming unrelated donors, a patient with constant CsA levels of 100 ng/mL had a 7%, 27%, and 66% higher hazard on days 10, 20, and 30 compared to a patient with constant CsA levels of 250 ng/mL over 100 days.

**FIGURE 2:**
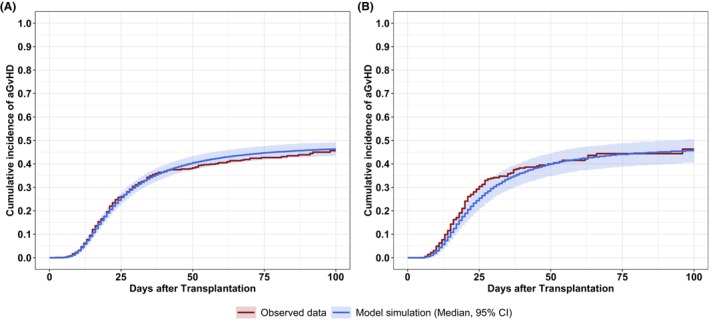
Model performance on the cumulative incidence of grades II–IV acute GvHD within 100 days after transplantation. (A) Descriptive performance of the final model on the training dataset. (B) Predictive performance of the final model on the test dataset. The red lines show the observed cumulative incidence. The blue lines show the model simulation. The blue shaded areas show the 95% confidence interval calculated from stochastic simulations of 1000 replicates.

In patients with related donors, patient age significantly affected the hazard elimination. This effect was estimated by an exponential model centred around 54 years with an estimated exponent of −0.97, reflecting that the hazard for younger patients decreased more rapidly than for older patients. For example, assuming constant CsA blood concentrations (250 ng/mL) and related donors, a 40‐year‐old patient had a 5%, 16%, and 30% lower hazard on days 10, 20, and 30 than a 54‐year‐old patient. Neither a significant effect of patient age in patients with unrelated donors nor of donor age nor the interaction of patient and donor age was detectable. The model (code provided in the supplement) described the training data adequately and predicted the test dataset well (Figure 2) with precisely estimated model parameters (RSEs <50%; Table 3). Additionally, parameter were stable as investigated by five‐fold cross validation (Table S7, Figure S2) with adequate performance metrics (5/5 models: log rank *p*‐value >0.10; Figure S3).

**TABLE 3:** Parameter estimates with relative standard error (%) of the final model.

Parameter	Value	RSE (%)
Basic model		
k_tr_	0.619	5.5
k_el_	0.0616	12.5
f	0.0339	10.4
Covariate effects		
$\gamma_{\mathrm{WBC}}$ ^a^	0.125	33.0
$\lambda_{\mathrm{MRD}}$ ^a^	0.773	13.2
$\lambda_{\mathrm{Sex}\;\text{mismatch}}$ ^a^	1.45	13.5
$\lambda_{\mathrm{Flu}}$ ^a^	0.768	12.7
$\gamma_{\mathrm{Age}}$ ^b^	−0.97	43.3
${\text{Slope}}_{\mathrm{CsA}}$ ^b^	0.00309	27.8

Abbreviations: CsA, cyclosporine A; f, scale parameter; k_tr_, hazard transit rate; k_el_, shape parameter (hazard elimination); RSE, relative standard error.

^a^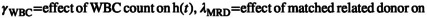 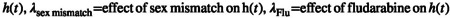 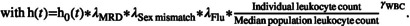

^b^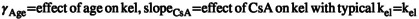 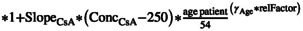; relFactor = 1 for related donors, relFactor = 0 for unrelated donors.

### 3.3 Stochastic simulation

#### 3.3.1 Visualization of risk factors

**FIGURE 3:**
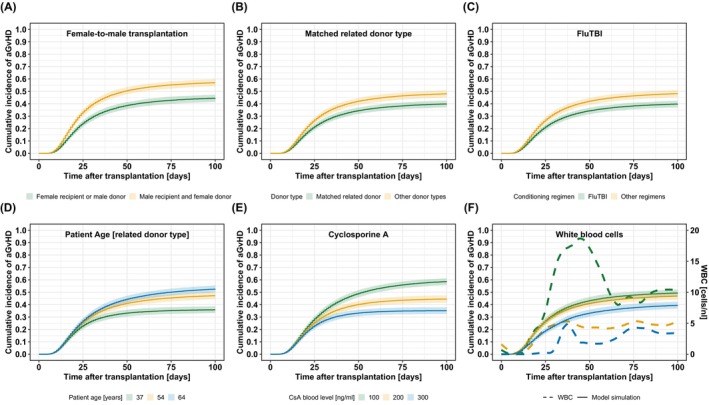
Simulation scenarios of covariate effects on grades II–IV aGvHD over 100 days after transplantation. (A–C) Stochastic simulations with and without covariate effect for the three discrete covariates: female donors for male recipients (A), matched related donors (B), and FluTBI conditioning regimen (C). (D) For the continuous covariate, patient age, three different age groups were simulated based on the 5th, 50th, and 95th percentile of the dataset: 37 years (green), 54 years (yellow), and 64 years (blue). (E) For the time‐dependent covariate, CsA blood level, three constant CsA blood levels of 100 ng/mL (green), 200 ng/mL (yellow), or 300 ng/mL (blue) were used. (F) For the time‐dependent covariate, WBC counts, three exemplary courses of leukocyte recovery over time from the training dataset were used for stochastic simulations: leukocytes recover early and strongly (green), early and moderate (yellow), and late and moderate (blue).

**FIGURE 4:**
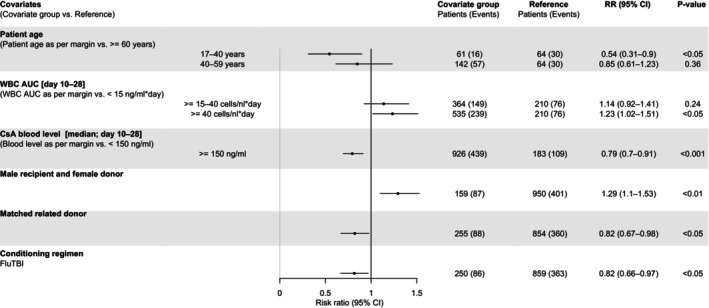
Adjusted risk ratios (95% CI) for covariate effects on grades II–IV aGvHD within 100 days after transplantation. Covariates that show a significant effect in the multivariable model were simulated univariate (1000 replicates) and risk ratio (black dot) and 95.0% confidence interval (error bar) for the survey period of 100 days after transplantation were calculated. The reference group for the age effect of unrelated donors were patients older than 59 years. To calculate RR for high, medium, and low WBC counts, the area under the WBC‐time curve (AUC) within days 10–28 was used as a surrogate since WBC counts strongly fluctuated over time after HSCT. The reference group for the WBC effect was WBC AUC below 15 cells/nl*days. The reference group for the CsA effect was CsA blood level below 150 ng/mL. The reference group for the female‐to‐male transplantation effect was transplantation of all other recipient‐donor gender combinations. The reference group for matched related donor effect was transplantation of all other donor types. The reference group for the FluTBI effect was the conditioning regimen of all other drug combinations. CsA, cyclosporine A, WBC, white blood cell.

Stochastic simulations were performed to demonstrate covariate effects on the cumulative incidence of grades II–IV aGvHD after HSCT (Figure 3). Here, assuming constant CsA blood levels, stochastic simulations revealed the non‐linear effect of CsA blood levels on the cumulative incidence of grades II–IV aGvHD (Figure 3E). Additionally, stochastic simulations of WBC counts showed, that an early and intense increase in WBC counts as well as an early and moderate increase, resulted in comparable cumulative incidences of grades II–IV aGvHD, unlike a late and moderate increase in WBC counts (Figure 3F). Adjusted RRs for the effects of covariates on the development of grades II–IV aGvHD within 100 days after HSCT were calculated (Figure 4). Patients with MRD, FluTBI treatment, and median CsA blood level above 150 ng/mL (days 10–28) showed significantly reduced risk. Moreover, the risk for younger patients with related donors was significantly reduced compared to peers over 60 years. Conversely, an increased risk was observed for patients with an area under the WBC‐time curve (AUC) above 40 cells/nl*days and male patients with female donors compared to all other recipient‐donor gender combinations.

#### 3.3.2 CsA blood level adaption

Stochastic simulations were conducted to illustrate potential adjustments required in the CsA blood level for patients with risk factors, aimed at achieving a cumulative incidence analogous to those patients without risk factors, maintaining a constant CsA blood level of 200 ng/mL (Figure S4).

**FIGURE 5:**
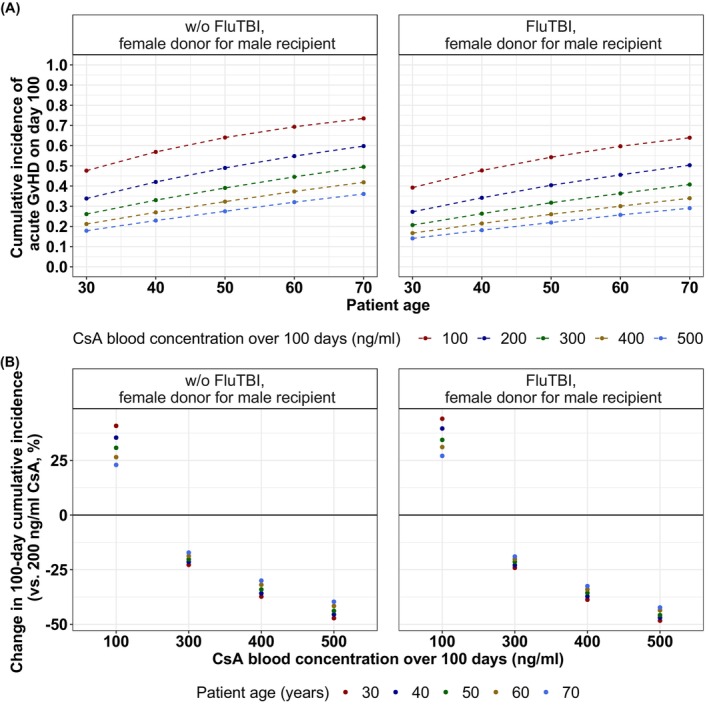
Median cumulative incidence of grades II–IV aGvHD on day 100 after transplantation for different ages and CsA blood concentrations stratified by FluTBI conditioning regimen in male recipients with female donors. (A) Median cumulative incidence of grades II–IV aGvHD on day 100 was simulated for five different constant CsA blood concentrations over 100 days (100, 200, 300, 400, 500 ng/mL) and five patient ages (30, 40, 50, 60, 70 years). (B) Estimated average percentage change in the 100‐day cumulative incidence for each stratum compared to a constant 200 ng/mL CsA blood concentration over 100 days. CsA, cyclosporine A, FluTBI, fludarabine plus total body irradiation.

To examine and visualize the relationship between CsA blood concentration and incidence of grades II–IV aGvHD, considering patient age, we plotted the simulated median 100‐day cumulative incidence of grades II–IV aGvHD against CsA blood concentration for discrete patient ages (Figure 5A). The figure demonstrates an approximately linear increase in 100‐day cumulative incidence as patient age rises. For example, at a CsA concentration of 200 ng/mL (dark blue points/line), the median cumulative incidence for a male patient with a female donor without FluTBI treatment increases 34% to 49% to 60% at ages 30, 50, and 70 years, respectively.

Furthermore, for higher CsA blood concentrations, the cumulative incidence over time decreases. The graph also reveals the increased CsA blood concentrations required for older patients to attain a similar incidence as younger patients with the same risk factors. For instance, the cumulative incidence for a 60‐year old male patient with a female donor without FluTBI conditioning regimen attaining 300 ng/mL CsA blood levels is comparable to a 30‐year old male patient with the same risk factors attaining 100 ng/mL CsA blood level (median; 60‐year‐old and 300 ng/mL CsA: 45% vs. 30‐year‐old and 100 ng/mL CsA: 48%).

Further analyses were performed to determine the potential reduction in the median 100‐day cumulative incidences of grades II–IV aGvHD achievable via alterations in constant CsA blood concentrations compared to a reference of 200 ng/mL (Figure 5B). For example, the 100‐day cumulative incidences for male patients with female donors of the ages of 30, 40, 50, 60 or 70 years, all receiving conditioning regimens other than FluTBI, would increase by 41%, 35%, 31%, 26%, and 23%, respectively, if CsA blood levels are reduced from 200 ng/mL to 100 ng/mL. For these patients, an increase in CsA blood levels from 200 ng/mL to 300 ng/mL, 400 ng/mL, or 500 ng/mL would result in reductions of the median cumulative incidences of 17–23%, 30–37%, or 40–47%, respectively. It is important to note that all simulations displayed in Figure 5 were conducted assuming constant CsA blood concentrations over a 100‐day duration.

### 3.4 Risk assessment day 7

In order to determine whether an early post‐allo‐HSCT time point can serve as an indicator for the later risk of developing grades II–IV aGvHD, we evaluated the correlation between an individual's cumulative hazard at selected time points (day 7, day 14, and day 21) and the subsequent number of observed events. For this, we classified patients into high and low‐risk categories based on their cumulative hazard at each time point relative to the population median. Stratification based on day 7 showed a marked distinction in event rate. In both the training and the test dataset, patients categorized as high‐risk exhibited higher incidence rates of grades II–IV aGvHD compared to those in the low‐risk group (training dataset: 41.41% vs. 34.52%, test dataset: 42.94% vs. 35.98%).

**FIGURE 6:**
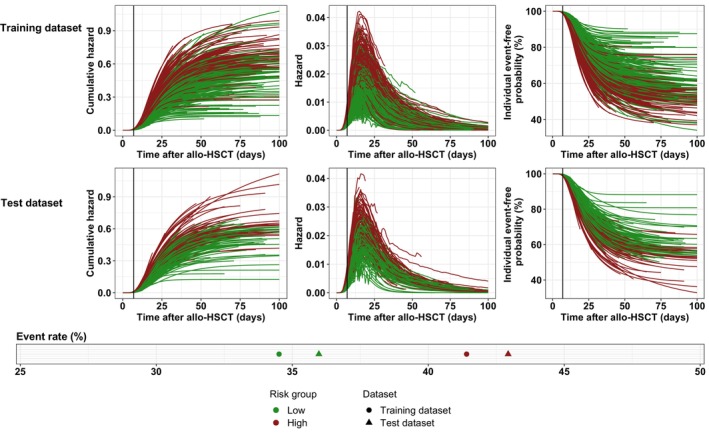
Individual time courses of cumulative hazard, hazard and event‐free probability stratified by test and training dataset. Each colored line represents an individual patient, black vertical lines indicate day 7 post‐HSCT. For each risk group the observed event rate within 100 days after allo‐HSCT was calculated (lower panel). Colors indicate risk group (red = high, green = low).

The described relationship is graphically depicted in Figure 6, where we plotted the cumulative hazard, current hazard, and individual probabilities of not developing grades II–IV aGvHD over time for each patient in both the test and training datasets. Additionally, the event rates for the two risk groups are presented, stratified by the respective dataset.

## 4 DISCUSSION

This study presents a novel dynamic TTE model for grades II–IV aGvHD, which identified a time‐varying Bateman function to modulate baseline hazard and investigate the role of CsA blood levels and WBC count as continuous longitudinal data without grouping. By applying the modeling approach of fitting joint longitudinal and time‐to‐event data, we gained deeper insight into the role of CsA blood levels and WBC count on the development of grades II–IV aGvHD. Additionally, multivariable analysis confirmed significant static factors (female donors for male recipients, MRD, FluTBI and patient age in subjects with related donors) for the development of grades II–IV aGvHD within 100 days after HSCT.

Elevated CsA blood levels were significantly associated with a lower risk for developing grades II–IV aGvHD. This finding is consistent with previous studies^14^, ^15^, ^16^, ^17^, ^18^, ^19^, ^20^, ^21^ that employed stratified data analysis^17^, ^18^, ^19^ or used varying cut‐off values for CsA concentrations (150 ng/mL,^14^ 195 ng/mL,^21^ 200 ng/mL,^34^ 348 ng/mL,^16^ 350 ng/mL^15^). In contrast, our study considered CsA exposure as a continuous, time‐dependent variable, providing a more fine‐grained concentration‐effect relationship. Previous studies have reported a 30% decrease in the incidence of aGvHD per 100 ng/mL increase in CsA blood levels. In the presented study, we observed a comparable but slightly smaller reduction in 100‐day incidence per 100 ng/mL, indicating a non‐linear relationship (Figure 5B). Notably, the reduction in incidence is more pronounced at lower CsA blood concentrations compared to higher levels.

Our stochastic simulations have demonstrated that the risk associated with static factors, such as age, might be mitigated by adjusting CsA blood levels. For this, necessary modulations of treatment with CsA can be effectively estimated using the presented model. However, formulating a precise CsA target level range that balances risk and benefit proves challenging due to ambiguous results concerning the correlation between CsA exposure and potential adverse outcomes (toxicities).^15^, ^35^, ^36^, ^37^ Various side effects associated with CsA usage, such as nephrotoxicity, neurotoxicity, hepatotoxicity and hypertension, have been documented.^38^ Notably, nephrotoxicity is most frequently reported, whereas neurotoxicity predominated in cases of acute overdose (>1000 ng/mL).^39^ However, the literature lacks definitive conclusions regarding the blood concentration‐toxicity relationship. While CsA trough levels exceeding 500 ng/mL are considered to induce nephrotoxicity,^40^, ^41^ this linkage has not been confirmed by other investigations.^15^, ^37^

Additionally, WBC count was found to be another longitudinal factor influencing the development of grades II–IV aGvHD. Specifically, a lower WBC count is associated with a reduced hazard. Interestingly, lymphocyte and neutrophil counts or the ratio of lymphocytes over granulocytes did not show a significant effect on hazard for grades II–IV aGvHD, despite the well‐established role of T‐cells in aGvHD development^33^, ^42^, ^43^ and the previous report of steep neutrophil recovery after allogeneic HCT as risk factor for aGvHD.^44^ The prediction of aGvHD development may be complicated by the composition of lymphocytes, which includes not only conventional T‐cells that contribute to aGvHD but also regulatory T‐cells and natural killer cells, both of which play a role in modulating the severity of aGvHD. This diversity in lymphocyte subtypes may interfere with the accuracy of predicting aGvHD based on undifferentiated lymphocyte counts. However, WBC, primarily composed of lymphocytes and neutrophils, exhibited a significant impact on hazard, with lower WBC counts associated with a higher hazard for developing grades II–IV aGvHD. It should be noted, that WBC counts were measured 1.9 times more frequently compared to lymphocyte and neutrophil counts, which may have biased the perceived importance of WBC count as a reliable biomarker compared to individual cell types in our model.

The effect of male recipients with female donors^4^, ^6^, ^7^ and the effect of MRD^6^, ^9^ on the hazard of developing grades II–IV aGvHD have been described in previous studies. However, the effect of male recipients with female donors could not be confirmed in all studies^9^ or only for certain severity grades.^12^ The presented analysis confirmed both factors. Our stochastic simulations indicate that female patients or patients with male donors attaining 200 ng/mL CsA blood levels over 100 days exhibited a median cumulative incidence of 42.7%. In contrast, male patients with female donors would require 62.5% higher CsA levels (325 ng/mL) for a comparable median incidence rate. Furthermore, patients with donor types other than MRD would require 37.5% higher CsA levels (275 ng/mL) over 100 days for a similar cumulative aGvHD incidence as patients with MRD attaining 200 ng/mL CsA blood levels over 100 days (median; MRD: 38.2% vs. non‐MRD: 38.9%).

For related‐donor patients, older patient age significantly increased the hazard of grades II–IV aGvHD. Although patient age is a commonly considered risk factor for aGvHD,^4^ conflicting results have been reported regarding its effect on aGvHD^12^ or its exclusive effect on chronic GvHD.^6^ To address potential confounders, we tested the effect of ATG treatment and specifically examined the interaction of highly correlated variables (recipient age and donor age) within the subgroup of related‐donor patients. However, no significant effects could be observed.

Patient age may affect the development of aGvHD by increasing thymic dysfunction and thereby impairing the negative selection of host‐reactive T‐cells, as supported by studies showing a correlation between low pre‐transplantation sjTREC counts and higher risk of aGvHD.^45^, ^46^ It is worth noting that a pediatric study previously reported a substantial decline in thymic activity among patients with MUD compared to those with MRD within the observation period of 6 months following HSCT.^47^ This suggests that the age of patients with MUD may play a less crucial role in influencing thymic function compared to those with MRD.

It is well‐known that aGvHD is initiated by tissue damage caused by myeloablative conditioning regimens,^33^, ^42^, ^43^ while reduced‐intensity conditioning regimens are assumed to provoke less aGvHD. Considering that the transplant conditioning intensity (TCI) score^48^ designates FluTBI based on the predominant cumulative dose applied (8 Gy: 52.2%, 10 Gy: 22.3%, 12 Gy: 24.0%) within the intermediate‐intensive conditioning regimens, the observed reduction in hazard for aGvHD seems reasonable.

The relationship between the patient‐individual cumulative hazard on day 7 and the actual number of observed events indicates a potential pathway for early‐stage patient risk classification, as exemplified in our analysis (see Figure 6). A potential clinical application of this risk classification could assist in identifying high‐risk patients by leveraging the estimated day 7 cumulative hazard. Consequently, this approach could guide clinicians in their decisions regarding the administration of escalated CsA dosages, aiming to optimize treatment outcomes.

The developed predictive model for aGvHD offers an approach for early detection and management of patients at risk, especially considering the notable incidence of steroid resistance in grades II–IV aGvHD cases. The model allows for timely interventions by estimating the probability of aGvHD development between allo‐HSCT and Day 100. Potential interventions include adjusting CsA blood levels or administering ATG to remove donor T cells. These strategies, guided by the model estimations, could contribute mitigating aGvHD progression and enhance patient management in clinical settings. Further research should explore the model's integration into clinical workflows and validate its applicability across varied patient groups.

In contrast to previous studies,^49^, ^50^, ^51^ our model was developed using a large dataset and incorporated a comprehensive combination of baseline pre‐HSCT parameters along with longitudinal laboratory results (such as WBC counts and CsA blood levels). Our approach differs from previous approaches that predominately relied on static variables,^51^, ^52^ utilized a limited set of covariates,^50^ or relied on data that is not readily available in routine clinical practice.^49^

However, our study has some limitations. Firstly, the dataset only provided information on the initial diagnosis date and the maximum grade of severity of aGvHD, which limited the ability to develop a predictor of initial severity and time from the initial diagnosis to the maximum grade of severity. Secondly, the analysis dataset, due to censoring, included a slightly smaller cumulative incidence of grades II–IV aGvHD at 38%, compared to the 41% observed in the uncensored raw data. Moreover, the data was obtained from a single centre, potentially introducing biases.^53^, ^54^ Furthermore, the model does not account for potential effects of tapering off immunosuppressive drugs, such as methotrexate or mycophenolate mofetil, as detailed information regarding their co‐administration (individual dose, duration, or measured plasma concentration) was unavailable. Additionally, our model does not account for potential interaction of WBC and various affection factors (infections, treatment with anti‐infection drugs, granulocyte colony‐stimulation or granulocyte‐macrophage colony‐stimulating factors, transfusion and poor graft function) due to unavailability of specific patient data. Lastly, the model relies on daily longitudinal data to predict the onset of aGvHD, which necessitates making assumptions or forecasts regarding individual daily WBC counts and CsA blood levels for the period of prospective investigation.

Despite these limitations, the primary strength of our model and analysis lies in its emphasis on capturing time‐depending changes following allo‐HSCT. Firstly, the estimated baseline hazard for developing grades II–IV aGvHD was modeled using a Bateman function, which represents periods after allo‐HSCT where the likelihood of an event is higher (around day 17) or lower (in the very first days after allo‐HSCT or temporally distant days). This allows for a more nuanced understanding of the temporal dynamics. Secondly, laboratory values such as CsA and WBC are included as longitudinal and continuous covariates, ensuring that no information is lost due to grouping or discretization.

In conclusion, we successfully developed a TTE model to predict the development of grades II–IV aGvHD within 100 days after HSCT. The results indicate that CsA treatment effectively reduces the cumulative incidence of grades II–IV aGvHD. However, to optimize CsA dosing using our model, further investigations are needed to explore CsA concentration‐dependent adverse effects and establish a clearer dose‐concentration relationship. In addition to confirming previously known factors influencing the development of aGvHD, we identified a negative effect of WBC counts on the hazard to develop grades II–IV aGvHD. However, it is important to note that further examination is required to distinguish the specific influences of lymphocyte and WBC counts, which would necessitate more frequent and finely‐grained observations, allowing for better stratification and analysis. Furthermore, the individual cumulative hazard on day 7 post‐allo‐HSCT demonstrates an association with the 100‐day incidence of grades II–IV aGvHD. This observation suggests that an assessment of the cumulative hazard at this early time point could serve as a useful predictor for the subsequent development of this condition.

## AUTHOR CONTRIBUTIONS

**Katharina Och:** Data curation (equal); formal analysis (equal); investigation (equal); visualization (equal); writing – original draft (equal); writing – review and editing (equal). **Amin T. Turki:** Investigation (equal); resources (equal); writing – review and editing (equal). **Katharina M. Götz:** Data curation (equal); writing – review and editing (equal). **Dominik Selzer:** Investigation (equal); supervision (equal); writing – review and editing (equal). **Christian Brossette:** Data curation (equal); software (equal). **Stefan Theobald:** Data curation (equal); software (equal). **Yvonne Braun:** Resources (equal). **Norbert Graf:** Funding acquisition (equal); software (equal). **Jochen Rauch:** Data curation (equal); software (equal). **Kerstin Rohm:** Data curation (equal); software (equal). **Gabriele Weiler:** Project administration (equal); software (equal). **Stephan Kiefer:** Project administration (equal); software (equal). **Ulf Schwarz:** Data curation (equal); software (equal). **Lisa Eisenberg:** Data curation (equal); writing – review and editing (supporting). **Nico Pfeifer:** Data curation (equal). **Matthias Ihle:** Software (equal). **Andrea Grandjean:** Software (equal). **Sonja Fix:** Software (equal). **Claudia Riede:** Software (equal). **Jürgen Rissland:** Investigation (equal); writing – review and editing (equal). **Sigrun Smola:** Resources (equal). **Dietrich W. Beelen:** Resources (equal); writing – review and editing (equal). **Dominic Kaddu‐Mulindwa:** Investigation (equal); writing – review and editing (equal). **Jörg Bittenbring:** Investigation (equal); writing – review and editing (equal). **Thorsten Lehr:** Conceptualization (lead); funding acquisition (equal); investigation (lead); resources (equal); supervision (equal); writing – review and editing (equal).

## FUNDING INFORMATION

The XplOit project was funded by the German Federal Ministry of Education and Research (grant number 031L0027A‐F).

## CONFLICT OF INTEREST STATEMENT

The authors declare the following competing interests: Thorsten Lehr received funding from Neovii Biotech. Amin T. Turki received consulting fees from CSL Behring, MSD, JAZZ Pharmaceuticals, and MaaT Pharma; travel subsidies from Neovii Biotech. Dietrich W. Beelen received travel subsidies from Medac. The remaining authors declare no competing financial interests.

## ETHICS STATEMENT

This study was approved by the institutional review board (IRB) of the medical association of the Saarland (Protocol N° 33/17) and the IRB of the University Duisburg‐Essen (Protocol N° 17‐7576‐BO). The requirement for written informed consent was waived due to the retrospective nature of the research and the use of anonymized data.

## Supporting information


Data S1.

## Data Availability

The used data includes sensitive personal health information. Due to the high dimensionality and inclusion of longitudinal data, datasets cannot be fully anonymized and published without the risk of re‐identification. Data access may be requested from the University Hospital Essen requiring approval by the data protection officer and ethics committee. To allow independent replication of our methods, the model code is provided in the Supplementary Materials.
