# Supplementary material for: A dynamic time‐to‐event model for prediction of acute graft‐versus‐host disease in patients after allogeneic hematopoietic stem cell transplantation
Source: Cancer Med. 2023 Dec 22;13(1):e6833. doi: 10.1002/cam4.6833 (PMC10807572; doi:10.1002/cam4.6833)
Supplement: Supplementary file 1 — Data S1. [file CAM4-13-e6833-s001.pdf]

Cancer Medicine

A dynamic time-to-event model for prediction of acute graft-versus-host disease in patients after allogeneic hematopoietic stem cell transplantation

Supplementary Material

Katharina Och[1], Amin T. Turki[2], Katharina M. Götz[1], Dominik Selzer[1], Christian Brossette[3], Stefan Theobald[3], Yvonne Braun[3], Norbert Graf[3], Jochen Rauch[4], Kerstin Rohm[4], Gabriele Weiler[4], Stephan Kiefer[4], Ulf Schwarz[5], Lisa Eisenberg[6], Nico Pfeifer[6], Matthias Ihle[7], Andrea Grandjean[7], Sonja Fix[7], Claudia Riede[7], Jürgen Rissland[8], Sigrun Smola[8,9], Dietrich W. Beelen[2], Dominic Kaddu-Mulindwa[10], Jörg Bittenbring[10], Thorsten Lehr[1*]

[1]Department of Clinical Pharmacy, Saarland University, Saarbrücken, Germany, [2]Department of Haematology and Stem Cell Transplantation, West-German Cancer Centre, University Hospital Essen, Essen, Germany, [3]Department of Pediatric Oncology and Haematology, Saarland University, Homburg, Germany, [4]Department of Biomedical Data & Bioethics, Fraunhofer Institute for Biomedical Engineering (IBMT), Sulzbach, Germany, [5]Institute for Formal Ontology and Medical Information Science, Saarland University, Saarbrücken, Germany, [6]Department of Computer Science, University of Tübingen, Tübingen, Germany, [7]Averbis GmbH, Freiburg, Germany, [8]Institute of Virology, Saarland University Medical Center, Homburg, Germany, [9]Helmholtz Institute for Pharmaceutical Research Saarland (HIPS), Helmholtz Centre for Infection Research (HZI), Saarland University Campus, Saarbrücken, Germany, [10]Department of Internal Medicine 1, University Hospital of the Saarland, Homburg, Germany

Corresponding Author:
Dr Thorsten Lehr, Department of Clinical Pharmacy, Saarland University,
Campus C5.3, 66123 Saarbrücken, Germany, Email: thorsten.lehr@mx.uni-saarland.de,
Phone: +49 681 302 70255, Fax: +49 681 302 70258

Funding:
This work is part of the XplOit (Semantic support for predictive modelling in systems medicine) project and was supported by grants from the German Federal Ministry of Education and Research (grant number 031L0027A-F).

Table of Contents

1 Supplementary Methods ... 3

1.1 Covariate model ... 3

1.2 Cross validation ... 4

1.3 Stochastic simulations ... 5

2 Supplementary Tables ... 6

Supplementary Table 1: Tested covariates for aGvHD time-to-event analysis. ... 6

Supplementary Table 2: Overview of conditioning regimens of the training and test datasets grouped by frequency of application and sorted alphabetically. ... 7

Supplementary Table 3: Used GvHD prophylaxis ... 8

Supplementary Table 4: Used ATG cumulative dosis – all patients ... 8

Supplementary Table 5: Used ATG cumulative dosis – depending on donor type ... 8

Supplementary Table 6: Used ATG cumulative dosis – depending on stem cell source ... 9

Supplementary Table 7: Parameter estimates with relative standard error (%) of the final model (training dataset) in comparison to the cross validation fold (sets 1–5) ... 10

3 Supplementary Figure ... 11

Supplementary Figure 1: Flowchart of the patient selection for model development. ... 11

Supplementary Figure 2: Parameters distribution of five-fold cross validation. ... 12

Supplementary Figure 3: Performance evaluation on the cumulative incidence of developing grades II–IV aGvHD by five-fold cross validation. ... 13

Supplementary Figure 4: Potential CsA adjustment for patient with risk factors to achieve comparable grade II–IV aGvHD cumulative incidence compared with patients without risk factors. ... 14

4 Supplementary NONMEM control stream ... 15

Final TTE Model ... 15

5 References ... 18

1 Supplementary Methods

1.1 Covariate model

Relevant covariates were gathered from previous publications on the development of aGvHD [1–9] or selected by exploratory analysis of covariate-stratified Kaplan-Meier curves using the existing dataset (Supplementary Table 1). We performed multivariable covariate analysis on the base model using a significance level of 5% and analyzed both longitudinal data of cell counts and CsA blood levels, as well as static data of patient characteristics, donor characteristics, and the HSCT procedure. Effects of covariates were either implemented by directly modulating the base hazard or the hazard function parametrization. Continuous covariates were centred around the population median and tested via linear or power functions. Missing values of patient characteristics were replaced by population mode for discrete variables and by population median for continuous variables. If more than one measurement per day and patient was available, the median was used. Missing longitudinal laboratory data were imputed using Last Observation Carried Forward (LOCF). If data was missing at the time of transplantation, we used Next Observation Carried Backward (NOCB). CsA whole blood concentrations were obtained during clinical routine and quantified via antibody-conjugated magnetic immunoassay (ACMIA), hence, measurement frequency varied between individual patients. To avoid confounding effects of other immunosuppressive drugs, patients that switched from CsA prophylaxis to another immunosuppressive drug were censored at the last time of CsA measurement. The conditioning regimens consisted of 32 different drug combinations (23% fludarabine, 22% fludarabine/treosulfan, 17% busulfan/fludarabine) with or without total body irradiation (TBI) resulting in 51 different conditioning regimens (see Supplementary Table 2). Fludarabine was always combined with TBI (2–12 Gy, FluTBI), fludarabine plus treosulfan was always administered without TBI, and busulfan plus fludarabine was primarily administered without TBI (99.6%, n=255/256). The effects of drug combinations and the use of TBI were tested separately.

1.2 Cross validation

To assess the final model regarding overfitting and stability, it was additionally evaluated by five-fold cross validation. For this, the complete dataset (n=1479) was split randomly into five equitably sized subgroups (subgroup 1–4: 296 IDs, subgroup 5: 295 IDs) using the R base function sample(). Subgroups were checked for comparable numbers of events (observed numbers of events: 113, 115, 118, 112 and 107 for subgroup 1–5, respectively). Subsequently, the parameters of the final model were re-estimated for each fold.

The parameter distributions of the five re-estimated parameter sets and the parameters of the final model were compared (see supplementary Figure 2). Here, parameter distribution were simulated based on the estimated parameters and the corresponding residual standard error (RSE) using the mvrnorm() function from the R package MASS.

For model assessment, performance metrics were obtained by simulating each of the five test datasets using the corresponding parameters sets and apply log-rank test for simulated vs. observed events. Subsequently an assessment of the model's performance was done by counting the number of significant log-rank tests results ($p<0.05$).

1.3 Stochastic simulations

To evaluate covariate effects on the cumulative incidence of grades II–IV aGvHD over time for 100 days after HSCT, we simulated 1000 replicates of the training dataset with the final TTE model. To isolate each covariate effect, all other covariates of the dataset were held constant. To examine the influence of covariates on the development of grades II–IV aGvHD within 100 days after transplantation, we calculated risk ratios (RRs) from simulations of 1000 replicates of the training dataset for each covariate, with the parameters of the other covariates set to “no effect”. For each discrete covariate, we divided simulations into a group containing individuals with the covariate (covariate group) and a group without the covariate (reference group) to calculate RRs. For continuous covariates, we grouped simulations by covariate cut-off values. To calculate RRs, we divided the proportion of individuals with an event in the covariate group by the proportion of individuals with an event in the reference group. Subsequently, we calculated the median, 2.5%, and 97.5% quantiles of all RRs.

2 Supplementary Tables

Supplementary Table 1: Tested covariates for aGvHD time-to-event analysis.

	**Covariate** Group	Subgroup	**Time-dependent†**	**Value type**
**Patients characteristics**	Patient age	-	no	numerical
	Diagnosis	Acute myeloid leukaemia	no	categorical
		Lymphoma	no	categorical
		Myelodysplastic syndromes + Myeloproliferative Disorder	no	categorical
	Disease stage‡	Advanced/early	no	categorical
**Donor characteristics**	Donor age	-	no	numerical
	Sex mismatch	Female-to-male HSCT	no	categorical
	Kinship (patient/donor)	related/unrelated	no	categorical
	HLA-matching§	matched/unmatched	no	categorical
	Donor type	matched unrelated donor	no	categorical
		matched related donor	no	categorical
		unmated unrelated donor		categorical
	Stem cell source	bone marrow/peripheral blood stem cells	no	categorical
**HSCT procedure**	Conditioning regimen (drug combination)	Fludarabine	no	categorical
		Fludarabine + Treosulfan	no	categorical
		Fludarabine + Busulfan	no	categorical
		Cyclophosphamide	no	categorical
	TBI	yes /no	no	categorical
	ATG	yes /no	no	categorical
**Laboratory value**	Medication	CsA blood level [ng/ml]	yes	numerical
	Cell count	Platelets [cells/nl]	yes	numerical
		Leukocytes [cells/nl]	yes	numerical
		Lymphocytes [cells/nl]	yes	numerical
		Basophils [cells/nl]	yes	numerical
		Eosinophils [cells/nl]	yes	numerical
		Neutrophils [cells/nl]	yes	numerical
		Monocytes [cells/nl]	yes	numerical

ATG: Anti-thymocyte globulin, TBI: Total body irradiation

†Time-dependent covariates were measured repeatedly and static covariates were assessed once before allo-HSCT.

‡Early stages: De-novo AML in 1st remission, ALL in 1st remission, MDS with single lineage dysplasia, and MDS with single lineage dysplasia and ring sideroblasts, CML in 1st chronic phase. Advanced disease stages: All other stages that did not correspond to early stages, such as AML in 2nd remission.

§HLA-matching: matched: 10/10 HLA-A-, -B, -C, -DRB1, -DQB1 match

Supplementary Table 2: Overview of conditioning regimens of the training and test datasets grouped by frequency of application and sorted alphabetically. In total, the XplOit dataset consists of 52 different conditioning regimens. Infrequently observed regimens with different cumulative doses of radiation were summarized.

**Conditioning Regimen**	**Training**		**Test**		**Complete**	
**Commonly used (>10%)**	Count	%	Count	%	Count	%
Busulfan + Fludarabine	194	17.49	61	16.49	255	17.24
Fludarabine + TBI (8 Gy)	132	11.9	44	11.89	176	11.9
Fludarabine + Treosulfan	231	20.83	91	24.59	322	21.77
**Occasionally used (1–10%)**						
Busulfan + Cyclophosphamide + Fludarabine	57	5.14	11	2.97	68	4.6
Carmustine + Fludarabine + Melphalan	102	9.2	18	4.86	120	8.11
Cyclophosphamide + TBI (12 Gy)	51	4.6	24	6.49	75	5.07
Cyclophosphamide + TBI (2–10 Gy)	48	4.33	15	4.05	63	4.27
Etoposide + TBI (8–12 Gy)	41	3.7	26	7.03	67	4.52
Fludarabine + Melphalan	19	1.71	3	0.81	22	1.49
Fludarabine + Melphalan + TBI (4–12 Gy)	37	3.33	10	2.7	47	3.19
Fludarabine + Melphalan + Treosulfan	24	2.16	7	1.89	31	2.1
Fludarabine + TBI (10 Gy)	53	4.78	22	5.95	75	5.07
Fludarabine + TBI (12 Gy)	60	5.41	21	5.68	81	5.48
**Rarely used (<1%)**						
Amsacrine + Cytarabine + Cyclophosphamide + Fludarabine + TBI (4–10 Gy)	3	0.27	2	0.54	5	0.34
Amsacrine + Cytarabine + Fludarabine + TBI (12 Gy)	3	0.27	0	0	3	0.2
Busulfan + Cyclophosphamide	2	0.18	0	0	2	0.14
Busulfan + Fludarabine + Melphalan	2	0.18	0	0	2	0.14
Busulfan + Fludarabine + TBI (8 Gy)	1	0.09	0	0	1	0.07
Carmustine + Etoposide + Melphalan	1	0.09	0	0	1	0.07
Carmustine + Fludarabine	1	0.09	0	0	1	0.07
Cyclophosphamide	6	0.54	1	0.27	7	0.47
Cyclophosphamide + Etoposide + TBI (10 Gy)	0	0	1	0.27	1	0.07
Cyclophosphamide + Etoposide + Treosulfan	10	0.9	3	0.81	13	0.88
Cyclophosphamide + Fludarabine	4	0.36	1	0.27	5	0.34
Cyclophosphamide + Fludarabine + TBI (2 Gy)	4	0.36	1	0.27	5	0.34
Cyclophosphamide + Melphalan + TBI (10 Gy)	0	0	1	0.27	1	0.07
Cyclophosphamide + Treosulfan	2	0.18	0	0	2	0.14
Cytarabine + Carmustine + Fludarabine + Melphalan	0	0	1	0.27	1	0.07
Cytarabine + Etoposide + TBI (12 Gy)	1	0.09	0	0	1	0.07
Cytarabine + Fludarabine + TBI (10–12 Gy)	2	0.18	0	0	2	0.14
Etoposide + Fludarabine + Treosulfan	1	0.09	0	0	1	0.07
Fludarabine + Melphalan + Thiotepa	3	0.27	1	0.27	4	0.27
Fludarabine + TBI (2 Gy)	4	0.36	0	0	4	0.27
Fludarabine + TBI (9 Gy)	0	0	1	0.27	1	0.07
Fludarabine + Thiotepa + TBI (8–12 Gy)	2	0.18	0	0	2	0.14
Fludarabine + Thiotepa + Treosulfan	1	0.09	1	0.27	2	0.14
Melphalan + TBI (8–12 Gy)	7	0.63	3	0.81	10	0.68

Supplementary Table 3: Used GvHD prophylaxis

	Training		Test		Complete	
	Count	%	Count	%	Count	%
CsA + MTX	909	81.97	307	82.97	1216	82.22
CsA + MTX + Prednisone/ solone	46	4.15	20	5.41	66	4.46
CsA + MMF	39	3.52	18	4.86	57	3.85
CsA + MTX + MMF	23	2.07	6	1.62	29	1.96
CsA + MTX + Tacrolimus	20	1.8	2	0.54	22	1.49
CsA + Prednisone/ solone + MMF	8	0.72	2	0.54	10	0.68
CsA	5	0.45	3	0.81	8	0.54
others	52	4.68	10	2.7	62	4.24

Supplementary Table 4: Used ATG cumulative dosis – all patients

	Training		Test		Complete	
**ATG dose [mg/kg]**	Count	%	Count	%	Count	%
0	497	44.82	170	45.95	667	45.1
30	480	43.28	155	41.89	635	42.93
60	89	8.03	36	9.73	125	8.45
others	43	3.88	9	2.43	52	3.52

Supplementary Table 5: Used ATG cumulative dosis – depending on donor type

		Training		Test		Complete	
**Donor type**	**ATG dose [mg/kg]**	Count	%	Count	%	Count	%
**Matched related**	0	241	94.51	85	95.51	326	94.77
	30	6	2.35	3	3.37	9	2.62
	60	1	0.39	0	0	1	0.29
	others	7	2.75	1	1.12	8	2.33
**Matched unrelated**	0	171	28.74	60	30.93	231	29.28
	30	348	58.49	119	61.34	467	59.19
	60	53	8.91	10	5.15	63	7.98
	others	23	3.87	5	2.58	28	3.55
**Unmatched unrelated**	0	83	33.6	24	28.92	107	32.42
	30	125	50.61	33	39.76	158	47.88
	60	29	11.74	23	27.71	52	15.76
	others	10	4.05	3	3.61	13	3.94
**Unmatched related**	0	2	16.67	1	25	3	18.75
	30	1	8.33	0	0	1	6.25
	60	6	50	3	75	9	56.25
	others	3	25.00	0	0	3	18.75

Supplementary Table 6: Used ATG cumulative dosis – depending on stem cell source

		Training		Test		Complete	
**Stem cell cource**	**ATG dose [mg/kg]**	Count	%	Count	%	Count	%
**Peripheral blood stem cell (PBSC)**	0	439	43.29	152	44.31	591	43.55
	30	458	45.17	151	44.02	609	44.88
	60	86	8.48	32	9.33	118	8.7
	others	31	3.06	8	2.33	39	2.87
**Bone marrow (BM)**	0	54	62.07	18	66.67	72	63.16
	30	20	22.99	4	14.81	24	21.05
	60	3	3.45	4	14.81	7	6.14
	others	10	11.49	1	3.7	11	9.65
**BM and PBSC**	0	4	50	0	0	4	50
	30	2	25	0	0	2	25
	60	0	0	0	0	0	0
	others	2	25	0	0	2	25

Supplementary Table 7: Parameter estimates with relative standard error (%) of the final model (training dataset) in comparison to the cross validation fold (sets 1–5)

Parameter	Training dataset		Cross validation (Set 1)		Cross validation (Set 2)		Cross validation (Set 3)		Cross validation (Set 4)		Cross validation (Set 5)	
	Value	RSE(%)	Value	RSE(%)	Value	RSE(%)	Value	RSE(%)	Value	RSE(%)	Value	RSE(%)
Basic model												
k_{tr}	0.619	5.50	0.636	5.3	0.625	5.2	0.618	5.0	0.648	5.5	0.621	5.0
k_{el}	0.0616	12.5	0.0599	12.4	0.0643	12.0	0.065	11.9	0.0607	11.9	0.0691	11.8
f	0.0339	10.4	0.0342	10.3	0.0345	10.1	0.0337	10.2	0.0339	10.0	0.0374	10.1
Covariate effects												
γ_{WBC}†	0.125	33.0	0.107	37.6	0.124	33.6	0.142	28.5	0.109	36.3	0.12	33.9
λ_{MRD}†	0.773	13.2	0.733	12.5	0.73	12.5	0.815	12.2	0.764	12.4	0.734	12.5
$\lambda_{sex\ mismatch}$†	1.45	13.5	1.38	13.2	1.56	12.5	1.39	13.1	1.5	13.6	1.49	12.8
λ_{Flu}†	0.768	12.7	0.829	11.8	0.862	11.6	0.805	12.0	0.806	11.8	0.804	11.9
γ_{Age}‡	-0.97	43.3	-0.731	47.7	-0.584	57.5	-0.864	43.3	-0.969	35.2	-0.859	43.1
$Slope_{CSA}$‡	0.00309	27.8	0.00262	33.9	0.00289	29.3	0.0036	21.0	0.003	29.0	0.00344	20.9

CsA: cyclosporine A, f: scale parameter, k_{tr}: hazard transit rate, k_{el}: shape parameter (hazard elimination), RSE: relative standard error

† γ_{WBC} = effect of WBC count on h(t), λ_{MRD} = effect of matched related donor on h(t), $\lambda_{sex\ mismatch}$ = effect of sex mismatch on h(t), λ_{Flu} = effect of fludarabine on h(t)

with $h(t) = h_0(t)*\lambda_{MRD}*\lambda_{Sex\ mismatch}*\lambda_{Flu}*\frac{\text{Individual leukocyte count}}{\text{Median population leukocyte count}}^{\gamma_{WBC}}$

‡ γ_{Age} = effect of age on k_{el}, $slope_{CsA}$ = effect of CsA on k_{el}

with typical $k_{el} = k_{el}*1+Slope_{CsA}*(Conc_{CsA}-250)*\frac{\text{age patient}}{54}^{(\gamma_{Age}*\text{relFactor})}$; relFactor = 1 for related donors, relFactor = 0 for unrelated donors

3 Supplementary Figure

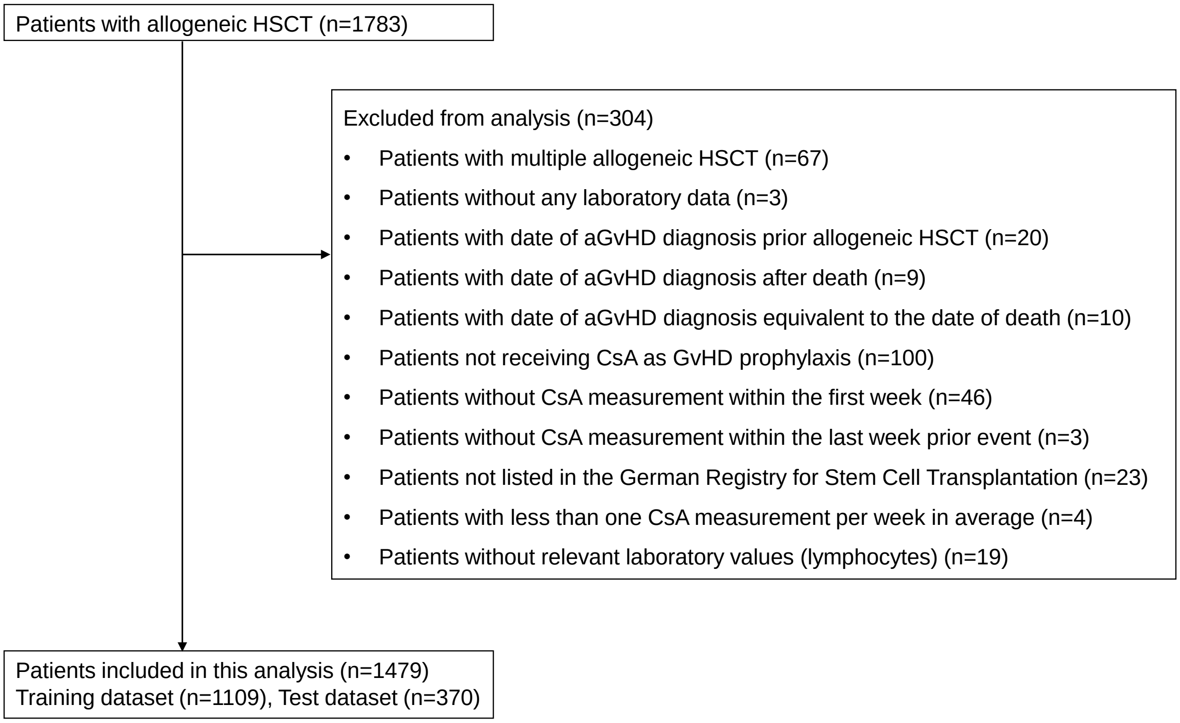

Supplementary Figure 1: Flowchart of the patient selection for model development.

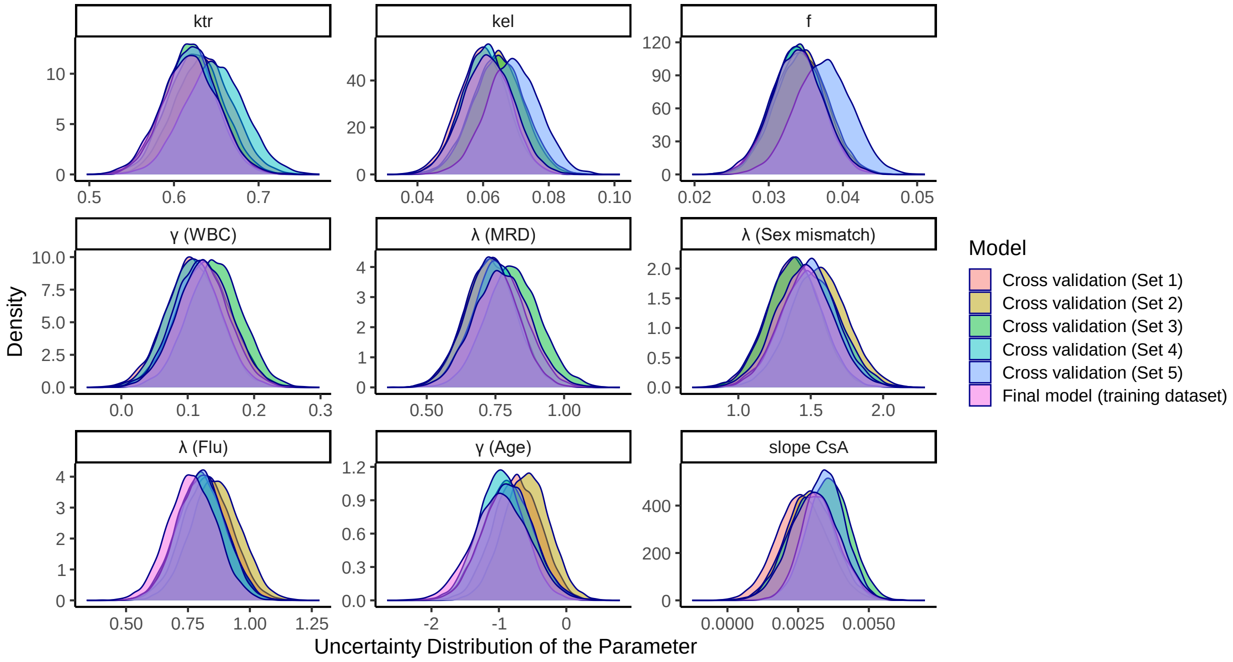

Supplementary Figure 2: Parameters distribution of five-fold cross validation.
Parameter distribution of estimated model parameters for each iteration of cross validation (sets 1–5) and for the final model were simulated and overlaid. Different colors represent different parameter sets.

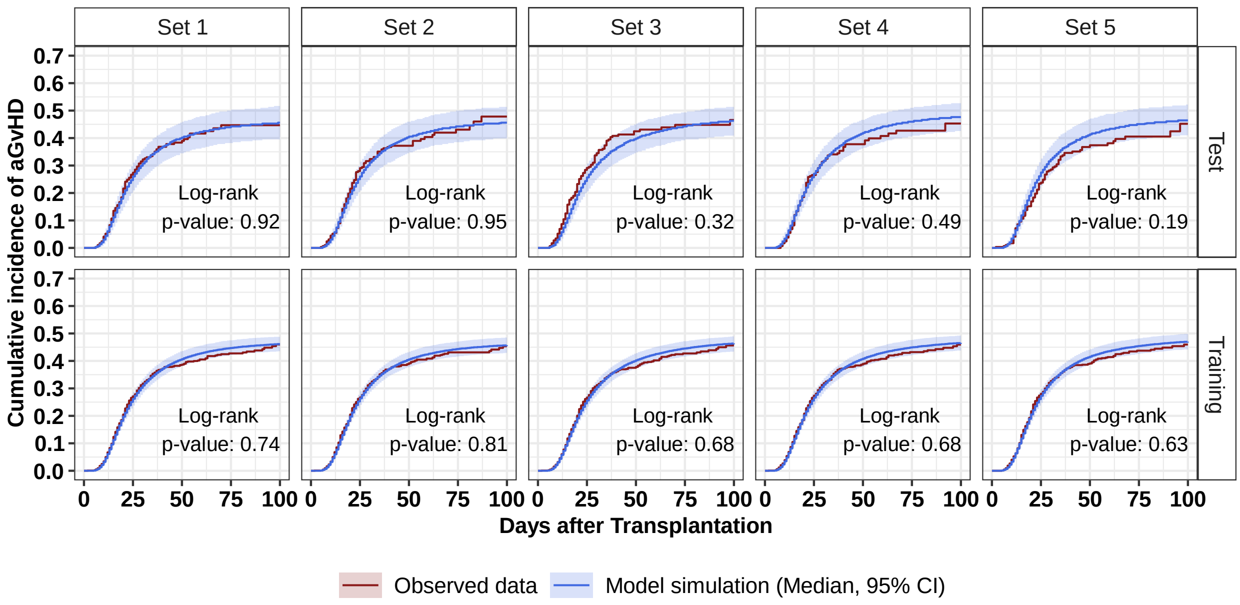

Supplementary Figure 3: Performance evaluation on the cumulative incidence of developing grades II–IV aGvHD by five-fold cross validation.

Model performance of the five iterations of cross validation process (sets 1–5) on the corresponding test (upper panel) and training dataset (lower panel). The red lines show the observed cumulative incidence. The blue lines show the median of model simulations. The blue shaded areas show the 95% confidence interval calculated from stochastic simulations of 1000 replicates.

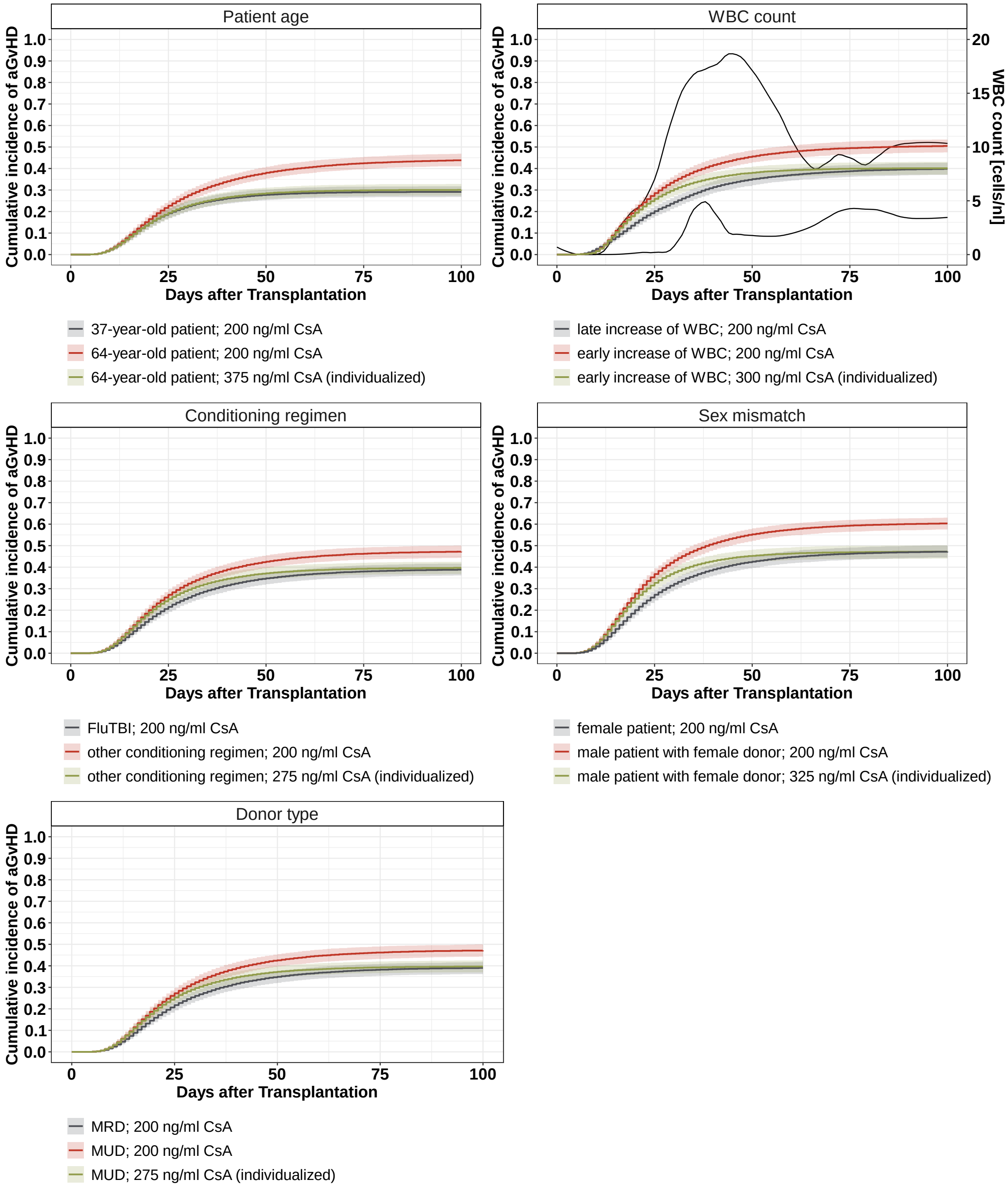

Supplementary Figure 4: Potential CsA adjustment for patient with risk factors to achieve comparable grade II–IV aGvHD cumulative incidence compared with patients without risk factors.

Simulation of grade II–IV aGvHD cumulative incidence were conducted for patients without risk factors (younger patients, patients with later increase in WBC, patients treated with FluTBI, patients with MRD and female patients; blue curve) and with the respective risk factor (red curve). CsA blood levels were assumed to be constant at 200 ng/ml for both groups. To match the incidences between the two groups, CsA blood levels were increased for the risk groups as indicated in the legend (green curve). CsA=cyclosporine A, FluTBI= fludarabine plus total body irradiation, MRD=matched related donor, MUD=matched unrelated donor, WBC=white blood cells

4 Supplementary NONMEM control stream

Final TTE Model

```
$SIZES NO=5000 PD=-100

; Sim_start: add to the simulation model

; $SIZES NO=500 LIM6=500

; Sim_end

$PROBLEM TTE SIM

$INPUT ID TIME AMT CMT EVID AGVHD_24=DV AGE_PAT AGE_DONOR SEX_PAT SEX_DONOR SEX_MATCH
STEMCELLSOURCE KINSHIP MACRIC_NO TBI_NO DIAG1_NO DISEASE_STAGE HLA_MATCH SPENDERGR FE
TYPE MDV BASOABS CSA_CONC EOSABS LEUKOSABS LYMPHOSABS MONOABS NEUTROABS THROMBOSABS
COND_COMBI TBI_YN ATG_YN LY_MED LEU_MED MONO_MED NEU_MED THR_MED EOS_MED BAS_MED


$DATA ../DATASET/AGVHD_TTE_ANALYIS.csv IGNORE=@

; Sim_start: remove from the simulation model

IGNORE=(TYPE.EQ.2)

; Sim_end


$SUBROUTINES ADVAN6 TOL=9


$MODEL

COMP(HAZARD)          ;1 Hazard

COMP(ABS)             ;2

COMP(CENTRAL)         ;3

COMP(TRANS1)          ;4 Transit 1

COMP(TRANS2)          ;5 Transit 2

COMP(TRANS3)          ;6 Transit 3

COMP(TRANS4)          ;7 Transit 4

COMP(TRANS5)          ;8 Transit 5

COMP(TRANS6)          ;9 Transit 6


$PK

IF (NEWIND.NE.2) TP=0

; Parameter Covariates

AGE_GA=0                               ; Y_Age for patients with unrelated donors

IF(KINSHIP.EQ.1)AGE_GA=THETA(6)        ; Y_Age for patients with related donors

AGE_HAZ=(AGE_PAT/54)**AGE_GA           ; Effect of patients age
```

```
SLOPE=THETA(7)                         ; SlopeCsA

CSA_HAZ= 1+SLOPE*(CSA_CONC-250)        ; Effect of CsA plasma concentration,
                                       ; median ConcCsA=250 ng/ml

KIN_HAZ= 1                             ; λ not MRD

IF(SPENDERGR.EQ.2)KIN_HAZ=THETA(3)     ; λ MRD

GA=THETA(4)                            ; γWBC
LEU_FACTOR=((LEUKOSABS+0.00001)/(LEU_MED+0.00001))**GA ; Effect of Leukocyte count

SEX_HAZ= 1                             ; λ not Sex mismatch

IF(SEX_MATCH.EQ.3)SEX_HAZ=THETA(8)     ; λ Sex mismatch

FLU_HAZ= 1                             ; λ not Flu

IF(COND_COMBI.EQ.1)FLU_HAZ=THETA(9)    ; λ Flu

; Parameter Base Model

ktr=THETA(1)*EXP(ETA(1))               ; Hazard transit rate,
                                       ; NONMEM requires one ETA (fixed to 0)

KE=THETA(2)*CSA_HAZ*AGE_HAZ            ; Shape parameter

F2=THETA(5)                            ; Scale parameter


$DES

DADT(2) = -ktr * A(2)

DADT(3) =  ktr * A(2) - KE* A(3)

DADT(1) =  A(9)*FLU_HAZ*SEX_HAZ*LEU_FACTOR*KIN_HAZ

DADT(4) =  ktr*A(3)-ktr*A(4)

DADT(5) =  ktr*A(4)-ktr*A(5)

DADT(6) =  ktr*A(5)-ktr*A(6)

DADT(7) =  ktr*A(6)-ktr*A(7)

DADT(8) =  ktr*A(7)-ktr*A(8)

DADT(9) =  ktr*A(8)-ktr*A(9)


$ERROR

HAZNOW=A(9)*FLU_HAZ*SEX_HAZ*LEU_FACTOR*KIN_HAZ       ; Hazard

IF(NEWIND.NE.2) OLDCHZ=0                             ; Initialization of OLDCHZ
                                                     ; for each ID

CHZ =A(1)-OLDCHZ                                     ; Cumulative hazard

; Sim_start: add/remove for simulation

; OLDCHZ=A(1)

IF(DV.NE.0) OLDCHZ=A(1)

; Sim_end

SUR=EXP(-CHZ)                          ; Survival function
```

```
IF(DV.EQ.0) Y=SUR                       ; Non-event DV=0
IF(DV.NE.0) Y=SUR*HAZNOW                ; Event DV=1
TP=TIME
; Simulation
IF(ICALL.EQ.4) THEN                     ; Only called for simulation
   CALL RANDOM (2,R)                    ; Generate a random number (between 0 and 1)
   DV=0
   IF(R.GT.SUR) DV=1
   RTTE = 0
   IF(TIME.EQ.100) RTTE = 1             ; Flag for censored observation at
                                        ; day +100 (end of observation)
   IF(R.GT.SUR) RTTE = 1                ; Flag for event
ENDIF

$THETA
(0, 0.619)          ; 1 ktr
(0, 0.0616)         ; 2 kel
(0, 0.773,2)        ; 3 λ MRD
(0, 0.125,1)        ; 4 γWBC
(0, 0.0339)         ; 5 f
(-2, -0.97)         ; 6 γAge
(0, 0.00309,1)      ; 7 SlopeCsA
(0, 1.45,3)         ; 8 λ Sex mismatch
(0, 0.768,3)        ; 9 λ Flu

$OMEGA
 0 FIX              ; Dummy variable

; Sim_start: add/remove for simulation
; $SIMULATION (5988566) (39978 UNIFORM) ONLYSIM NOPREDICTION SUB=100
$ESTIM MAXEVAL=9990 METHOD=1 LAPLACE LIKE PRINT=1 MSFO=msfb999 SIGL=9 NSIG=3 NOABORT
$COV PRINT=E
;Sim_end
$TABLE ID TIME DV KINSHIP EVID HAZNOW CHZ SUR NOPRINT ONEHEADER FILE=sdtab515
```

5 References

1. Holler E, Greinix H, Zeiser R. Acute Graft-Versus-Host Disease. In: Carreras E, Dufour C, Mohty M, Kröger N, editors. The EBMT Handbook: Hematopoietic Stem Cell Transplantation and Cellular Therapies. Cham (CH): Springer International Publishing; 2019. page 323–30.

2. Anasetti C, Beatty PG, Storb R, Martin PJ, Mori M, Sanders JE, et al. Effect of HLA incompatibility on graft-versus-host disease, relapse, and survival after marrow transplantation for patients with leukemia or lymphoma. Hum. Immunol. 1990;29:79–91.

3. Flowers ME, Inamoto Y, Carpenter PA, Lee SJ, Kiem HP, Petersdorf EW, et al. Comparative analysis of risk factors for acute graft-versus-host disease and for chronic graft-versus-host disease according to National Institutes of Health consensus criteria. Blood 2011;117:3214–9.

4. Nash RA, Pepe MS, Storb R, Longton G, Pettinger M, Anasetti C, et al. Acute graft-versus-host disease: Analysis of risk factors after allogeneic marrow transplantation and prophylaxis with cyclosporine and methotrexate. Blood 1992;80:1838–45.

5. Weisdorf D, Hakke R, Blazar B, Miller W, McGlave P, Ramsay N, et al. Risk factors for acute graft-versus-host disease in histocompatible donor bone marrow transplantation. Transplantation 1991;51:1197–203.

6. Kollman C, Howe CWS, Anasetti C, Antin JH, Davies SM, Filipovich AH, et al. Donor characteristics as risk factors in recipients after transplantation of bone marrow from unrelated donors: The effect of donor age. Blood 2001;98:2043–51.

7. Jagasia M, Arora M, Flowers MED, Chao NJ, McCarthy PL, Cutler CS, et al. Risk factors for acute GVHD and survival after hematopoietic cell transplantation. Blood 2012;119:296–307.

8. Lee SE, Cho BS, Kim JH, Yoon JH, Shin SH, Yahng SA, et al. Risk and prognostic factors for acute GVHD based on NIH consensus criteria. Bone Marrow Transplant. 2013;48:587–92.

9. Zeiser R, Blazar BR. Acute Graft-versus-Host Disease — Biologic Process, Prevention, and Therapy. N. Engl. J. Med. 2017;377:2167–79.
